# Berberine Poisoning with Polymorphic Ventricular Tachycardia: A Case Report

**DOI:** 10.5811/cpcem.56949

**Published:** 2026-08-05

**Authors:** Melson P Mesmin, Marit S Tweet, Sharon H Kim, Tyler J Fulks

**Affiliations:** Southern Illinois University School of Medicine, Department of Emergency Medicine, Springfield, Illinois

**Keywords:** berberine, polymorphic ventricular tachycardia, supplements, QTc prolongation, case report

## Abstract

**Introduction:**

Natural supplements are readily available without a prescription and are not regulated by the United States Food and Drug Administration. The popularity of berberine, a bioactive compound used for centuries in traditional Chinese medicine, has surged due to its proposed benefits in glycemic control and cardiovascular health. However, use of berberine may lead to possible negative electrophysiologic changes.

**Case Report:**

A 92-year-old male presented to the emergency department (ED) with chief complaints of tremors, urinary incontinence, and brief syncopal episodes that began approximately two weeks after starting berberine. In the ED he had multiple episodes of polymorphic ventricular tachycardia with pulselessness requiring immediate cardioversion, with a rapid return of spontaneous circulation between episodes. Amiodarone was started; however, despite this, he continued to have persistent episodes. Finally, the episodes resolved after starting lidocaine and isoproterenol in the intensive care unit. The patient’s workup was notable for heart rate-corrected QT interval (QTc) prolongation (QTc 616 milliseconds (ms) [male reference range, QTc < 430 ms]) confirming the polymorphic ventricular tachycardia as torsades de pointes. His cardiac evaluation was otherwise normal, including a normal ejection fraction on a transthoracic echocardiogram. Withholding berberine and supportive management resulted in an improved QTc. On hospital day 3, the QTc was 454 ms. The patient had a short inpatient admission and was discharged without any further episodes.

**Conclusion:**

Berberine has numerous effects and is purported to have beneficial effects and cardioprotective properties. Due to induced bradycardia and QT prolongation, patients who take berberine can be vulnerable to life-threatening arrhythmias, such as the R-on-T phenomenon in our patient’s case. Additional research on berberine is needed to better inform clinician–patient discussions about its use.

## INTRODUCTION

Many natural supplements are readily available without a prescription; their safety and any claims of health benefits are not regulated by the U.S. Food and Drug Administration. Berberine, a bioactive compound extracted from the plant *Coptis chinesis*, has been used for centuries in traditional Chinese medicine. Recent interest in berberine has surged due to its proposed benefits in glycemic control and cardiovascular health.^1^ Berberine is readily available as an over-the-counter supplement in many pharmacies and is marketed for a variety of indications.^2^,^3^ Although generally considered safe, emerging case reports suggest berberine may exert significant pharmacologic and toxicologic effects with potential for adverse cardioactive outcomes such as predisposition to developing a dysrhythmia.^4^,^5^ This case report demonstrates a case in which a patient implemented berberine to manage his diabetes and presented with heart rate-corrected QT interval (QTc) prolongation and polymorphic ventricular tachycardia.

## CASE REPORT

A 92-year-old man presented to the emergency department (ED) with complaints of brief unresponsive episodes. His past medical history included type 2 diabetes mellitus and hypertension. The patient’s daily medication included 500 mg of metformin twice daily, 25 mg of empagliflozin, 2.5 mg lisinopril, and 81 mg of aspirin. Additionally, he supplemented his prescription medication regimen with daily ingestion of 500 mg of berberine, 1,000 mg of vitamin C, and 30 mg of zinc. Notably, he began the berberine supplement approximately two weeks prior to his presentation to the ED. The patient’s supplement use was not known at the time of his ED presentation.

On initial presentation, the patient was alert and oriented to person, time, and place. His initial vital signs were as follows: heart rate, 61 beats per minute; blood pressure, 200/64 mm Hg; respiratory rate, of 18 breaths per minute; oral temperature, 36.5 °C; and oxygen saturation, 98% on room air. His physical examination was unremarkable. While on telemetry, the patient developed polymorphic ventricular tachycardia with pulselessness. Immediate cardiopulmonary resuscitation with rapid cardioversion resulted in return of spontaneous circulation. The patient suffered an additional episode of pulseless ventricular tachycardia shortly thereafter, which was again corrected with rapid cardioversion. A 12-lead electrocardiogram showed normal sinus rhythm with a QTc of 616 ms, confirming the polymorphic ventricular tachycardia as torsades de pointes (Image). The patient was reflexively started on amiodarone (150 mg intravenous [IV] bolus followed by 1 mg/minute infusion) in accordance with Advanced Cardiac Life Support guidelines for the management of shockable cardiac arrest.^6^

Lab testing was significant only for an elevated white blood cell count of 21 cells/μL (reference range, 4–10 cells/μL). The remainder of the patient’s lab testing including complete metabolic panel, serum magnesium, high sensitivity troponin and thyroid stimulating hormone levels were all within normal range. The chest radiograph did not reveal any acute cardiopulmonary process.

*CPC-EM Capsule*

What do we already know about this clinical entity?

*Berberine can prolong the correct QT interval (QTc), increasing risk of bradycardia and torsades de pointes, though it is often considered a “safe” supplement.*

What makes this presentation of disease reportable?

*Recurrent pulseless polymorphic ventricular tachycardia due to berberine-related QTc prolongation in an elderly patient was initially unrecognized due to undisclosed supplement use.*

What is the major learning point?

*Unregulated supplements may contribute to life-threatening dysrhythmias; thorough histories must include over-the-counter medications and herbal agents.*

How might this improve emergency medicine practice?

*Early recognition of supplement-induced QT prolongation helps guides appropriate.*

The patient did not have further episodes in the ED and was admitted to the intensive care unit for further monitoring. Immediately with clinical stability, a review of the patient’s ED course identified the episode to be related to the prolonged QTc interval. Amiodarone was discontinued due to its QTc prolongation effects. The patient was started on lidocaine (100 mg IV push followed by 1 mg/minute infusion) for the next 24 hours. He additionally received a 2 g magnesium sulfate IV bolus to abet further episodes of polymorphic ventricular tachycardia. After the lidocaine, he was administered isoproterenol (2 mcg/minute IV infusion) for a total of 16 hours, and ventricular tachycardia episodes ceased. The cardiology team was consulted, and a cardiac workup included a transthoracic echocardiogram which showed an ejection fraction of 70–75% (reference range, 55–75%) and did not show structural abnormalities or valvulopathy.

Ultimately the cardiology team determined berberine supplementation was the most likely cause of the QTc abnormalities and recurrent ventricular tachycardia. Withholding berberine while hospitalized resulted in QTc interval improvement from 616 ms to 454 ms (reference range, < 430 ms). After normalization of the QTc interval and cessation of all cardiac infusions, the patient was discharged home on hospital day 4 with instructions to discontinue berberine and follow up with primary care and cardiology in the outpatient setting.

## DISCUSSION

Berberine has been associated with electrophysiologic effects, including sinus bradycardia and prolongation of the QTc interval. These effects are primarily attributed to its inhibition of the cardiac human ether-à-go-go-related gene channels, which play a crucial role in cardiac repolarization.^7^ Inhibition of these channels can lead to delayed repolarization and QT interval prolongation, increasing the risk dysrhythmias such as torsades de pointes.^5^,^7^ In this case, the patient developed episodes of polymorphic ventricular tachycardia shortly after initiating berberine in the setting of baseline bradycardia. Berberine has been shown to have a negative chronotropic effect, decreasing the frequency of spontaneous contractions in cardiac pacemaker cells. This bradycardic effect, combined with QTc prolongation, can create a high-risk dysrhythmic state.^8^

The cardiology consultants attributed the dysrhythmia to a R-on-T phenomenon, likely triggered by the combination of slow sinus rhythm and delayed repolarization. This mechanism is consistent with previously described pathways for torsades de pointes in which early afterdepolarizations during a prolonged QT interval can initiate a potentially fatal tachyarrhythmia.^9^ The dysrhythmogenic potential of berberine remains under-recognized, particularly given its widespread availability as an over-the-counter supplement marketed for cardiovascular and metabolic health. This case illustrates a paradox in pharmacologic modulation, in which therapies intended to treat cardiac conditions may, if misapplied or insufficiently monitored, precipitate life-threatening dysrhythmias.

## CONCLUSION

This case highlights the potential for serious cardiotoxic effects associated with berberine, a widely available herbal supplement. While berberine is often promoted for its cardiometabolic benefits, clinicians should recognize its potential to cause significant cardiac adverse effects. Greater vigilance is warranted in assessing supplement use during clinical evaluation, particularly in patients with cardiovascular comorbidities. This case underscores the need for further research into the electrophysiologic safety profile of berberine and reinforces the importance of patient counseling regarding the risks of unregulated supplements.

## Figures and Tables

**Image.**
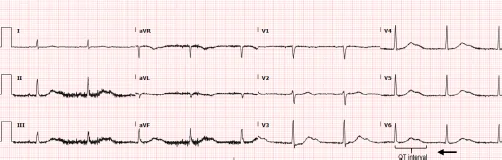
Patient’s electrocardiogram demonstrating prolonged QT interval shown by the arrow in a patient with berberine toxicity.
